# Land Use Alters the Drought Responses of Productivity and CO_2_ Fluxes in Mountain Grassland

**DOI:** 10.1007/s10021-017-0178-0

**Published:** 2017-09-15

**Authors:** Johannes Ingrisch, Stefan Karlowsky, Alba Anadon-Rosell, Roland Hasibeder, Alexander König, Angela Augusti, Gerd Gleixner, Michael Bahn

**Affiliations:** 1 Institute of Ecology, University of Innsbruck, Sternwartestraße 15, 6020 Innsbruck, Austria; 2 Max Planck Institute for Biogeochemistry Jena, Postbox 100164, 07701 Jena, Germany; 3 Department of Evolutionary Biology, Ecology and Environmental Sciences, University of Barcelona, Avinguda Diagonal 643, 08028 Barcelona, Spain; 4 Institute of Agro-Environmental and Forest Biology, CNR Italy, Via G. Marconi n.2, 05010 Porano, TR Italy

**Keywords:** Carbon cycle, Climate extreme, Gross primary productivity, Land-use change, Nitrogen, Recovery, Resilience, Resistance, ^15^N labelling

## Abstract

Climate extremes and land-use changes can have major impacts on the carbon cycle of ecosystems. Their combined effects have rarely been tested. We studied whether and how the abandonment of traditionally managed mountain grassland changes the resilience of carbon dynamics to drought. In an in situ common garden experiment located in a subalpine meadow in the Austrian Central Alps, we exposed intact ecosystem monoliths from a managed and an abandoned mountain grassland to an experimental early-summer drought and measured the responses of gross primary productivity, ecosystem respiration, phytomass and its components, and of leaf area index during the drought and the subsequent recovery period. Across all these parameters, the managed grassland was more strongly affected by drought and recovered faster than the abandoned grassland. A bivariate representation of resilience confirmed an inverse relationship of resistance and recovery; thus, low resistance was related to high recovery from drought and vice versa. In consequence, the overall perturbation of the carbon cycle caused by drought was larger in the managed than the abandoned grassland. The faster recovery of carbon dynamics from drought in the managed grassland was associated with a significantly higher uptake of nitrogen from soil. Furthermore, in both grasslands leaf nitrogen concentrations were enhanced after drought and likely reflected drought-induced increases in nitrogen availability. Our study shows that ongoing and future land-use changes have the potential to profoundly alter the impacts of climate extremes on grassland carbon dynamics.

**Electronic supplementary material:**

The online version of this article (doi:10.1007/s10021-017-0178-0) contains supplementary material, which is available to authorized users.

## Introduction

The frequency and severity of extreme climatic events are expected to increase in the near future, with major implications for the carbon (C) cycle of ecosystems and related feedbacks to the atmosphere and the climate system (Reichstein and others 2013; Frank and others 2015). On a global scale, severe droughts are amongst the climate extremes exerting the strongest effects on the C cycle (Ciais and others 2005; Reichstein and others 2013; Knapp and others 2015). The overall resilience of an ecosystem to climate extremes can be characterized by the resistance, that is, the ability of an ecosystem to persist during disturbance, and the recovery, that is, the ability of a system to return to pre-disturbance levels (Holling 1996; Hodgson and others 2015; Oliver and others 2015). Thus, a system may be resilient due to a high resistance, a high capacity to recover or both. Although the relationships and potential trade-offs between the components of resilience have been subject to recent discussions (for example, Hodgson and others 2015; Yeung and Richardson 2016), few studies have actually tested such relationships on the C cycle responses of ecosystems to drought.

Grasslands cover more than one-fifth of the global land surface (35 × 10^6^ km^2^, Dixon and others 2014) and constitute an important carbon sink (Conant and others 2001; Smith 2014). In many mountain regions of Europe, grasslands also play an important role in the production of fodder. In recent decades, land-use changes have led to the abandonment of mountain meadows and pastures (for example, MacDonald and others 2000; Tasser and others 2007), with consequences for species composition (Tasser and Tappeiner 2002), productivity and ecosystem C fluxes (Schmitt and others 2010), soil C and nitrogen (N) turnover (Zeller and others 2000; Robson and others 2007; Meyer and others 2012a; Grigulis and others 2013) and the water cycle (Obojes and others 2015). To date, surprisingly few studies have explored how land-use changes affect ecosystem responses to climate extremes (Bahn and others 2014). Although management intensity has been suggested to modify grassland responses of productivity and CO_2_ fluxes to precipitation variability and drought (Klumpp and others 2011; Vogel and others 2012; Zwicke and others 2013), the consequences of an abandonment of managed grasslands for the drought and post-drought responses of their C dynamics are largely unknown.

Site fertility has been shown to modify grassland responses to climate change (Grime and others 2000); however, the role of nutrient availability for ecosystem recovery from drought is still poorly understood. Recent studies suggest that soil nitrogen (N) dynamics can be altered by drought events and that rewetting of soil after drought can enhance N mineralization and consequently lead to higher tissue N concentrations (Fuchslueger and others 2014; Canarini and Dijkstra 2015; Arfin Khan and others 2016; Roy and others 2016). Increased leaf N concentrations can in turn promote photosynthetic C assimilation and thereby speed up ecosystem recovery from drought (Roy and others 2016). To date it is not known whether abandonment of mountain grasslands, associated with a reduction in N availability (Zeller and others 2000; Robson and others 2007), alters the role of plant N uptake during post-drought recovery, and what the consequences are for tissue N concentrations and CO_2_ uptake dynamics.

We established an experiment testing whether and how abandonment of managed mountain grassland changes the resilience of C dynamics to an extreme early-summer drought. We analysed both drought resistance and post-drought recovery of ecosystem CO_2_ fluxes and of the phytomass and its components. To understand the relationships and potential trade-offs between the components of resilience in response to land-use change, we applied a recently proposed bivariate representation of resilience (Nimmo and others 2015) and tested if the conclusions were robust across the different parameters studied. We furthermore applied a perturbance-based approach (Potts and others 2006; Todman and others 2016) to obtain an integrated quantification of the overall perturbation of the two grasslands by the drought event. Our main hypothesis was that abandonment increases the resistance of C dynamics to and decreases their recovery from drought. We furthermore tested the hypothesis that drought enhances N uptake and tissue N concentrations in managed grassland, whereas the role of plant N uptake during recovery is strongly reduced in abandoned grassland.

## Material and Methods

### Study Site

The study site is located near Neustift in the Stubai valley in the Austrian Central Alps and is composed of grasslands differing in land use, including a traditionally managed hay meadow and an abandoned grassland (Schmitt and others 2010). The two subalpine grasslands are located on a southeast exposed hillside with similar inclination (ca. 20°), average annual temperature (3°C), annual precipitation (1097 mm) and the same soil type (dystric cambisol). The soil textural fractions for clay, silt and sand are 13.3, 36.2 and 50.2%, respectively, on the meadow, and 23.4, 45.5 and 31.1%, respectively, on the abandoned grassland (Meyer and others 2012b). The meadow (1820–1850 m a.s.l.; 47°07′45″N, 11°18′20″E) is cut once per year at peak biomass in early August and is fertilized with manure every 2–3 years. Additionally, light grazing by sheep and cattle takes place in spring and late autumn. The vegetation community has been classified as *Trisetetum flavescentis* and consists of perennial grasses and forbs dominated by *Agrostis capillaris, Festuca rubra, Ranunculus montanus, Trifolium pratense and T. repens* (Bahn and others 2009). The second grassland (1970–2000 m a.s.l.; 47°07′31″N, 11°17′24″E) has been abandoned since 1983. Its vegetation has been classified as *Seslerio*-*Caricetum* with some dwarf shrubs and is dominated by *Sesleria varia, Erica carnea, Carex sempervirens* and *Poa alpina* (Schmitt and others 2010; Grigulis and others 2013). Further details concerning vegetation and soils, as well as the overall nutrient supply and productivity of the two sites, can be taken from Bahn and others (2006), Schmitt and others (2010), Meyer and others (2012a), Grigulis and others (2013), Fuchslueger and others (2014) and Legay and others (2014).

### Experimental Set-up

The drought experiment was conducted in a common garden established at the meadow site (see above). At both the meadow and abandoned grassland, 20 intact vegetation–soil monoliths were extracted in June 2013. The monoliths had a diameter of 25 cm and a height of 28 cm and were fit in open-top round stainless steel cylinders (height 40 cm), with a reservoir for leachates at the bottom (for detailed description see Obojes and others 2015). The monoliths were installed in the common garden in a randomized factorial design with six blocks and were left for almost a year before the drought experiment started in May 2014.

**Figure 1:**
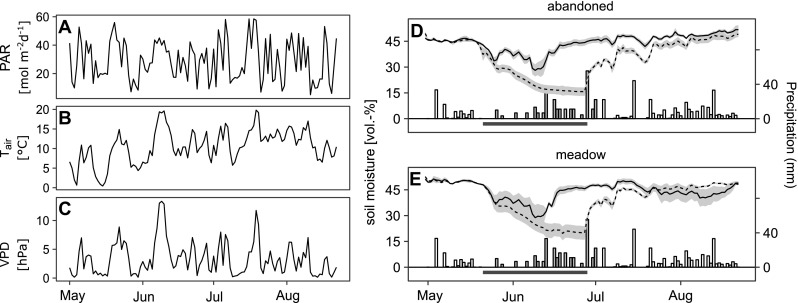
Time course of **A** daily sums of photosynthetically active radiation (PAR), daily means of **B** air temperature (*T*_air_) and **C** air vapour pressure deficit (vpd) and in the rain-out shelters. **C**, **D** Daily means of soil moisture (vol.%) in the main rooting horizon in monoliths from the meadow (control *n* = 3, drought *n* = 5) and the abandoned grassland (control *n* = 4, drought *n* = 5) exposed to ambient conditions (control, *solid line*) and drought (*dashed line*). *Shaded areas* show the standard error of the mean. *Vertical bars* show daily precipitation (*open* = natural,* shaded* = manual watering). Note that during rain exclusion (*horizontal black bar*) only monoliths from the control treatment received water.

For the drought experiment, each of the six blocks was covered with a rain-out shelter, which had a base area of 3 × 3.5 m^2^ and was open at the bottom (up to 0.5 m above ground) and at the top of the face sides to allow air circulation. Rain exclusion was performed with transparent UV-A and UV-B transmissive plastic foil (Lumisol clear AF, Folitec, Westerburg, Germany, light transmittance c. 90%). The rain exclusion lasted from 21 May 2014 until 28 June 2014. During this period, monoliths allocated to the drought treatment did not receive any precipitation. Control monoliths were manually watered every 1–4 days with previously collected rainwater to maintain soil water content above 25 vol.% (Figure 1D, E). The amount of water added to the controls was 180 and 170 mm for the meadow and the abandoned grassland, respectively. On 28 June 2014, rain-out shelters were removed and 50 mm of rainwater was added to each of the monoliths to simulate a heavy rain event ending the drought, and to achieve a well-defined and rapid rewetting.

### Measurements

#### Microclimate

A microclimate station at the common garden recorded photosynthetically active radiation (PAR), precipitation, air temperature and humidity (see details in Hasibeder and others 2015). During the rain exclusion, air temperature, humidity and PAR (S-THB-M002 and S-LIA-M003, onset Computer Corporation, Bourne, MA, USA) were additionally measured in two of the six rain-out shelters. Soil water content (Decagon EC-5, 5TM, 5TE; combining SWC and temperature, logger Em50; Decagon Devices, Pullman, WA, USA) and soil temperature (sensors S-TMB, logger HOBO Micro Station H21-002; Onset Computer corporation, Bourne, MA, USA) were measured continuously in the main rooting horizon (30-min interval) in a subset of the monoliths (SWC: *n* = 17, Temp: *n* = 14) over the whole course of the season. In early May, before the start of the experiments, all soil moisture sensors were offset-calibrated in situ after a rainy period, when all monoliths had reached field capacity. To determine the water balance, we measured the amount of leachates accumulated in the reservoir of each monolith over the period of rain exclusion (Obojes and others 2015). Total evapotranspiration during the drought experiment was estimated for the subset of monoliths equipped with soil moisture sensors by means of a water balance approach, accounting for the amount of water added, the change of water storage in each monolith as derived from monitored changes in volumetric soil water content in the main rooting horizon, and the amount of leached water (Table S1; see also Obojes and others 2015).

#### Phytomass

Aboveground phytomass was sampled destructively during three campaigns, at peak drought (“resistance campaign” on 1 July 2014, *n* = 12, that is, 3 replicates per land use and treatment combination) and twice during the recovery period, that is, 4 (“recovery 1 campaign” on 24 July, *n* = 12) and 8 weeks (“recovery 2 campaign” on 22 August, *n* = 16) after termination of the experimental drought. Thus, at each campaign, a separate subset of monoliths was harvested by cutting phytomass to 2 cm aboveground. The samples were frozen at −18°C until further analysis.

Phytomass samples were split into four functional groups (forbs, grasses, legumes, dwarf shrubs), and into stems, leaves, reproductive organs and living phytomass (hereafter biomass) and necromass (senesced plant parts) were separated. For each functional group in each monolith, specific leaf area (SLA) was obtained for a subset of leaves saturated with water and scanned (V700 Photo, Epson, WinRHIZO Pro 2012, Regent Instruments) and subsequently dried at 60°C for 3 days. Leaf area index (LAI) was calculated from the leaf biomass and SLA for each functional group. Community-weighted mean (CWM) of SLA was calculated as the leaf-biomass-weighted mean of SLA of each functional group.

#### Tissue Nitrogen and ^15^N Labelling

For each sampling campaign (see above), we measured the leaf nitrogen concentration (LNC) and its nitrogen isotope ratio (δ^15^N) on a subset of leaves sampled in each of the monoliths. Furthermore, at the end of the drought experiment, we performed a ^15^N pulse labelling experiment on the 12 monoliths sampled during the Recovery 1 campaign. 20 mg of KNO_3_ with 10% ^15^N (2 mg ^15^N per monolith) dissolved in 100 ml rain water was distributed equally on the soil of the monoliths. During the subsequent harvest, both the shoots and the roots from the uppermost 7 cm of the soil were sampled. Roots were extracted with a soil corer (3 cm diameter), washed, sieved to 2 mm and microwaved before transporting to the laboratory.

All plant samples were oven-dried at 60°C, ground, weighed (2–5 mg and analysed on an elemental analysis—isotope ratio mass spectrometer (EA-IRMS; EA 1100, CE Elantech, Milan, Italy; coupled to a Delta + IRMS, Finnigan MAT, Bremen, Germany). LNC was calculated based on the peak area and the known nitrogen concentration of external acetanilide standards. The δ^15^N was determined in per mil (%) relative to the international reference standard AIR-N_2_ using IAEA-N1 (Werner and Brand 2001). The amount of ^15^N label recovered in roots and shoots is calculated as:

$$ {\text{incorporated}}\; {}_{{}}^{15} {\text{N}} = \frac{{\left( {{\text{atom}}\%_{\text{labelled}} - {\text{atom}}\%_{\text{NA}} } \right)*{\text{N}}_{\text{pool}} }}{100 \% } $$

with atom%_labelled_ being the ^15^N atom% of the labelled samples, atom%_NA_ being the ^15^N atom% of natural abundance samples and N_pool_ being the respective nitrogen pool (mg N m^−2^).

#### CO_2_ Fluxes

We measured the net ecosystem exchange (NEE) of CO_2_ using closed dynamic chambers, similar to the system applied by Schmitt and others (2010). The chambers were transparent Plexiglas cylinders (diameter 25 cm, height 50 cm) which fitted airtight on the steel cylinders containing the monoliths. Pressure effects on CO_2_ fluxes were avoided by a hole in the top of the chamber, which was closed with a plug after placing the chamber. Air inside the chambers was ventilated with fans. Concentrations of CO_2_ (GMP 343, Vaisala Helsinki, Finland) and water vapour, as well as temperature (HMP 75, Vaisala, Helsinki Finland) were logged for 1 min with 5-s intervals. During each measurement, the photosynthetically active radiation (PQS1 PAR Quantum Sensor, Kipp & Zonen, Delft, the Netherlands) was recorded. Ecosystem respiration (ER) was measured by covering the chamber with a dark cloth, excluding any light inside the chamber. To obtain estimates of gross primary productivity (GPP), paired measurements of NEE under sunlit and dark conditions were taken. Monoliths were measured in randomized order in the morning hours on days with clear sky. In addition to sunlit and dark measurements, a series of light response curves were obtained for each treatment type, using layers of semitransparent cloth (Schmitt and others 2010). To obtain a consistent time series of CO_2_ fluxes throughout the whole study, flux measurements were taken on the monoliths which were harvested during the last campaign (Recovery 2).

CO_2_ flux rates were calculated as described by Schmitt and others (2010). Each measurement was quality controlled based on visual inspection and quality of the linear fits as recently recommended by Pirk and others (2016). GPP was calculated as the difference of the corresponding NEE and ER measurements. Throughout this study, GPP and ER fluxes are both assigned positive signs. For each land-use type and precipitation treatment, light response curves were obtained from pooled data by fitting a rectangular hyperbolic model (Ruimy and others 1995; Schmitt and others 2010). Above a photon flux density (PFD) of 1000 µmol m^−2^ s^−1^, all light response curves levelled off and reached 80–85% of the maximum values (Figure S1). Thus, for the sake of comparability across treatments we only present data obtained at PFD above this threshold and apply the term light-saturated GPP (GPP_sat_). For our analysis, we only included fluxes from measurement days for which at least three quality-controlled replicate data sets per land use and treatment combination were available.

### Calculations of Indices and Statistics

To obtain normalized fluxes, values of GPP_sat_ and ER in the drought treatment were divided by their respective values in the controls. The daily means of the normalized fluxes define the response trajectory of each grassland in the bivariate space of normalized GPP_sat_ and normalized ER. The Euclidian distance between consecutive measurement points is a measure of change within this bivariate space, and the cumulative length of each trajectory is a measure of the overall perturbation (Potts and others 2006).

The resistance of GPP_sat_, ER, leaf area index (LAI) and biomass was determined based on the measurements during peak drought. We express resistance (RST) as the ratio of drought to control measurements (Kaufman 1982). A recovery index according to Nimmo and others (2015) was calculated for GPP_sat_, ER, LAI and biomass. This index is a measure of the post-drought change of the parameter. We adapted the approach by using measurements of control monoliths instead of pretreatment measurements to account for seasonal changes in the controls. The recovery index was calculated as D_rec_/C_rec_ – RST, where D_rec_ and C_rec_ denote parameter values during recovery in the drought and control treatment, respectively, and RST is the resistance of the parameter (see above).

All calculations and statistical analyses were performed in R 3.2.3 (R Development Core Team 2015). We used permutational ANOVA with the package lmPerm (Wheeler 2010). For each sampling day, we tested for the interaction of land-use type and drought treatment and drought effects within each land-use type.

## Results

Key meteorological variables during the experimental period are presented in Figure 1A–C. During the rain exclusion, soil moisture declined to less than 20 vol.% in the monoliths from both grasslands which were exposed to drought (Figure 1 D, E). The drought treatment reduced the total amount of evapotranspiration significantly (*p* < 0.001) in both grasslands (Table S1). Neither land use nor the interaction of drought and land use had a significant effect on the evapotranspiration.

### Aboveground Plant Productivity and Nitrogen Relations

**Figure 2:**
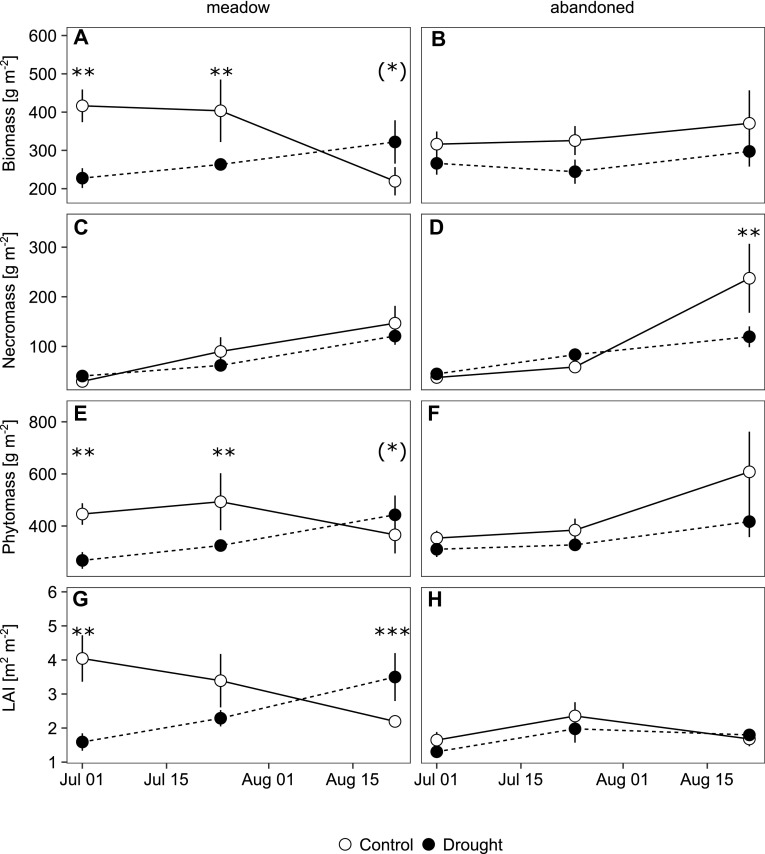
**A**–**F** Biomass, necromass, phytomass (sum of biomass and necromass) and **G**, **H** leaf area index (LAI) of monoliths from the meadow and the abandoned grassland subjected to ambient conditions (*open symbols*) and drought (*closed symbols*) and sampled at the end of drought (1 July) and during recovery (24 July, 22 August). *Error bars* indicate standard errors of the mean (*n* = 3 for July and *n* = 4 for August samplings), and *stars* indicate significant treatment effects within land use and sampling date (*p* value: *** < 0.001 < ** < 0.01 < * < 0.05 < (*) < 0.1).

The meadow had a generally higher biomass and leaf area index (LAI) compared to the abandoned grassland, but these differences declined in the course of the season (Figure 2A, B, G and H, Figure S3), reflecting a delayed development and lower aboveground net primary production of the abandoned grassland and an earlier plant senescence of the meadow plants (Figure 2C, D). During the period of peak growth in early July, leaf area index (LAI) was higher on the meadow compared to the abandoned grassland. The fraction of grasses was significantly higher in the abandoned grasslands (78 ± 3%) compared to the meadow (55 ± 5%, *p* < 0.001). The community-weighted mean (CWM) of SLA for the meadow and the abandoned grassland was 14.4 ± 0.8 and 9.3 ± 0.6 m^2^ kg^−1^, respectively (*p* < 0.001). Forbs had a significantly higher mean SLA in the meadow (20.3 ± 1.5 m^2^ kg^−1^) compared to the abandoned grassland (15.2 ± 1.5 m^2^ kg^−1^, *p* < 0.001), whereas the mean SLA of grasses did not differ significantly (meadow: 10.1 ± 1.3 m^2^ kg^−1^, abandoned grassland: 7.3 ± 0.8 m^2^ kg^−1^).

**Figure 3:**
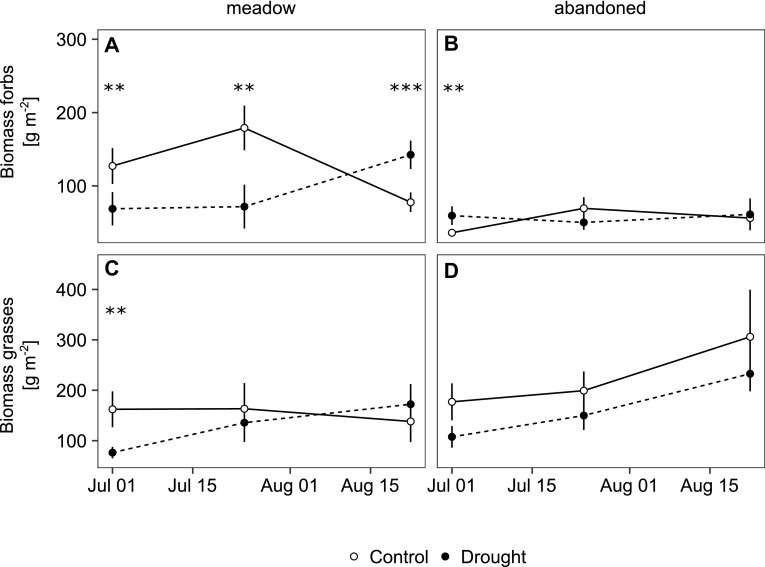
Biomass of **A**, **B** forbs and **C**, **D** grasses of monoliths from the meadow and the abandoned grassland under ambient conditions (*open symbols*) and drought conditions (*closed symbols*), sampled at the end of the drought treatment (1 July) and during recovery (24 July, 22 August). *Error bars* indicate standard errors of the mean (*n* = 3 in July and *n* = 4 in August), *asterisks* indicate significant treatment effects within land use and sampling date (*p* value: *** < 0.001 < ** < 0.01).

Drought reduced biomass significantly in the meadow (Figure 2A), but not in the abandoned grassland (Figure 2B, Table 1; Figure S2 and Tables S2 and S4). The drought-induced reduction was persistent for 4 weeks, but disappeared later when biomass in the control monoliths declined (Figure 2A). Drought did not immediately induce leaf senescence at either site, but significantly reduced necromass in the abandoned grassland in the late season (Table 1, Figure 2C, D). LAI was reduced by drought in the meadow (Table 1, Figure 2G, H), but exceeded the values in the controls at the Recovery 2 sampling. This was also reflected by leaf mass dynamics (Figure S2). Biomass of forbs responded differently between the two grasslands (Table 1, Figure 3A, B, Figure S3) and was less resistant but recovered more rapidly in the meadow. In contrast, biomass of grasses was significantly reduced by drought and recovered quickly in both grasslands (Table 2, Figure 3C, D, Figure S3).

**Table 1:** Drought and Land-Use Effects on Phytomass Parameters

	Campaign	Weeks after rewetting	Treatment	Land use	Treatment * land use
Biomass	Resistance	0	***	n.s.	*
	Recovery 1	3.5	***	n.s.	n.s.
	Recovery 2	8	n.s.	n.s.	n.s.
Necromass	Resistance	0	*	n.s.	n.s.
	Recovery 1	3.5	n.s.	n.s.	*
	Recovery 2	8	n.s.	n.s.	n.s.
Phytomass	Resistance	0	***	n.s.	*
	Recovery 1	3.5	***	n.s.	n.s.
	Recovery 2	8	n.s.	n.s.	n.s.
LAI	Resistance	0	***	***	**
	Recovery 1	3.5	*	(*)	n.s.
	Recovery 2	8	*	***	n.s.
Biomass of forbs	Resistance	0	n.s.	**	*
	Recovery 1	3.5	**	**	*
	Recovery 2	8	*	**	*
Biomass of grasses	Resistance	0	***	n.s.	n.s.
	Recovery 1	3.5	n.s.	n.s.	n.s.
	Recovery 2	8	n.s.	(*)	n.s.
LNC	Resistance	0		n.s.	n.s.
	Recovery 1	3.5	***	(*)	n.s.
	Recovery 2	8	*	n.s.	(*)
δ15N_NA_	Resistance	0	n.s.	***	n.s.
	Recovery 2	8	n.s.	***	n.s.

*Results of permutational ANOVA testing overall treatment effect, land-use effect and their interaction on different phytomass parameters. Biomass* *=* *living phytomass; Necromass* *=* *senescent phytomass; LAI* *=* *leaf area index; LNC* *=* *leaf nitrogen concentration; δ15N*_*NA*_ *=* *natural abundance nitrogen isotope ratio of leaves. Resistance* *=* *1 July, peak drought, Recovery 1* *=* *24 July, Recovery 2* *=* 22 August. Stars indicate the significance level: *** *<* *0.001* *<* ** *<* *0.01* *<* * *<* *0.05* *<* (*) *<* *0.1*

**Table 2:** Drought and Land-Use Effects on CO_2_ Fluxes

	Date	Days after rewetting	Treatment	Land use	Treatment * land use	Meadow	Abandoned
GPP_sat_	Pretreatment		n.s.	(*)	n.s.	n.s.	n.s.
	Resistance		***	***	*	***	***
	Recovery 1	5	**	n.s.	n.s.	*	***
	Recovery 2	9	*	(*)	n.s.	n.s.	(*)
	Recovery 3	17	n.s.	n.s.	n.s.	(*)	n.s.
ER	Pretreatment		n.s.	n.s.	n.s.	n.s.	n.s.
	Resistance		**	(*)	n.s.	**	*
	Recovery 1	5	**	n.s.	n.s.	***	*
	Recovery 2	9	n.s.	n.s.	n.s.	*	n.s.
	Recovery 3	17	n.s.	n.s.	*	n.s.	*
	Recovery 4	30	n.s.	n.s.	n.s.	n.s.	n.s.

*Results of permutational ANOVA testing overall treatment effect, land-use effect and their interaction as well as within-land-use effects of drought on key dates of experiment. GPP*_*sat*_ *=* *light-saturated rate of gross primary productivity, ER* *=* *ecosystem respiration. Dates: Pretreatment* *=* *19 May, Resistance* *=* *26 June, Recovery 1* *=* *3 July, Recovery 2* *=* *7 July, Recovery 3* *=* *15 July, Recovery 4* *=* 28 July. Stars indicate the significance level: *** *<* *0.001* *<* ** *<* *0.01* *<* * *<* *0.05* *<* (*) *<* *0.1*

**Figure 4:**
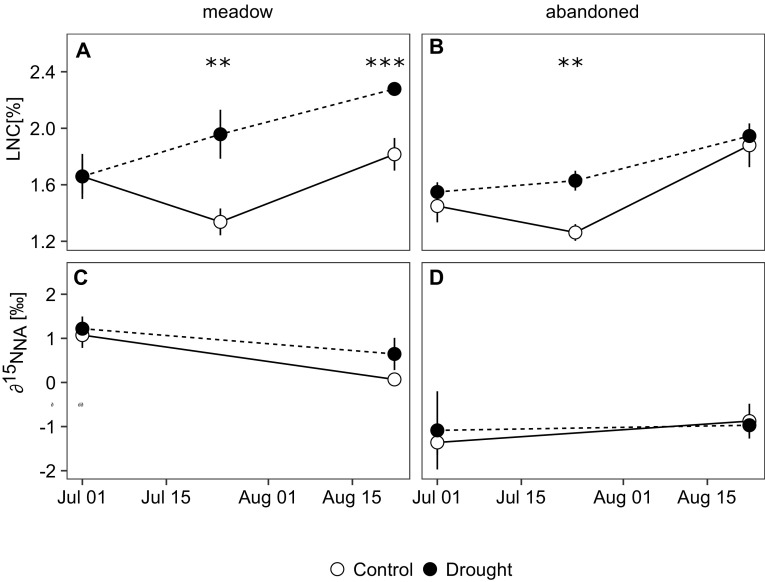
**A**, **B** Leaf nitrogen concentrations (LNC) and **C**, **D** corresponding natural abundance δ^15^N isotope values of leaves in monoliths from the meadow and the abandoned grassland subjected to ambient conditions (*open symbols*) and drought (*closed symbols*) and sampled at peak drought (1 July) and during recovery (24 July, 22 August). *Error bars* indicate standard errors of the mean (*n* = 3 for July and *n* = 4 for August samplings), *stars* indicate significant treatment effects within land use and sampling date (*p* value: *** < 0.001 < ** < 0.01 < * < 0.05).

During the recovery period, LNC was significantly higher in monoliths previously exposed to drought compared to the controls. The effect was observed in both grasslands and was more pronounced in the meadow (Table 1, Figure 4A, B). Leaf δ^15^N was generally higher in the meadow than in the abandoned grassland (Table 1, Figure 4C, D) and was not affected by drought.

**Figure 5:**
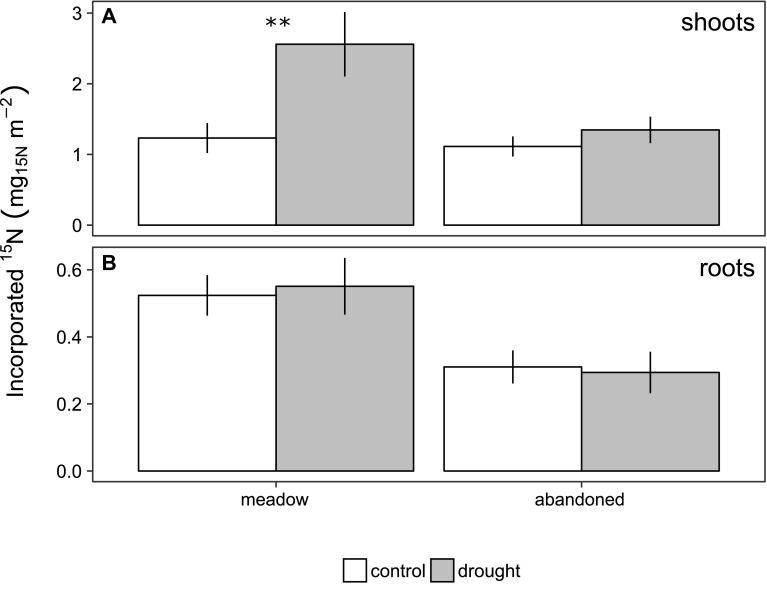
Amount of ^15^N label recovered in the two grasslands in **A** shoots and **B** roots of control monoliths (*open bars*) and in monoliths recovering from drought (*shaded bars*) 3 weeks after the rewetting. *Error bars* indicate standard errors of the mean (n = 3). *Stars* indicate significant differences between control and drought treatment (*p* value: ** < 0.01).

Following ^15^N pulse labelling meadow plants recovering from drought took up significantly more ^15^N label than the controls (*p* < 0.01) and incorporated this nitrogen into shoots (+110%, *p* < 0.01), but not into roots (Figure 5). In contrast, the abandoned grassland did not increase its uptake of ^15^N during the recovery from drought (Figure 5).

### CO_2_ Fluxes

**Figure 6:**
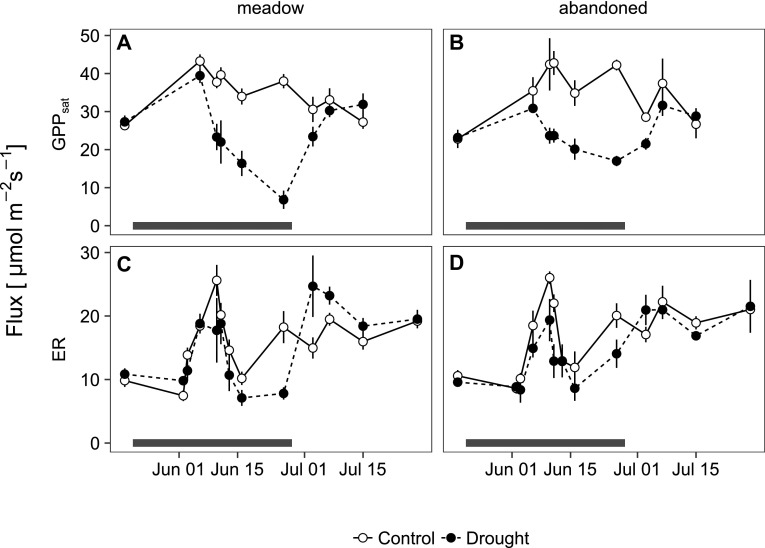
**A**, **B** Light-saturated rates of gross primary productivity (GPP_sat_) and **C**, **D** ecosystem respiration (ER) in monoliths from the meadow and the abandoned grassland subjected to ambient conditions (*open symbols*) and drought (*closed symbols*). *Error bars* indicate standard errors of the mean (*n* = 3–4). The *horizontal black bars* indicate the time of rain exclusion.

During drought, GPP_sat_ was progressively reduced to 20 and 40% of the controls in the meadow and the abandoned grassland, respectively (Figure 6A, B); the interaction of land use and drought was significant (Table 2). At peak drought, ER was reduced by up to 60 and 25% on the meadow and the abandoned site, respectively (Figure 6C, D); however, the interaction of land use and drought was not significant. After rewetting, GPP_sat_ fully recovered within 9 days in both grasslands (Table 2). Its recovery rate was distinctly higher in the meadow compared to the abandoned grassland (23.4 versus 14.6 μmol m^−2^ s^−1^ over the period of 9 days). During the recovery, ER was significantly enhanced in previously drought-exposed monoliths relative to controls in both grasslands, the effect being more pronounced and sustained in the meadow than in the abandoned grassland (Figure 6C, D; Table 2).

### Effects of Land-Use Change on Integrated Drought Responses

**Figure 7:**
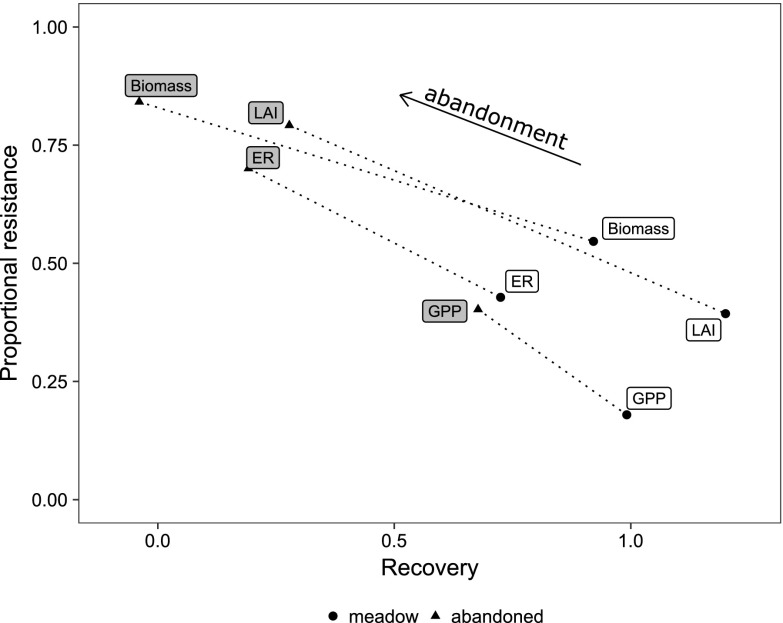
Resistance and recovery of the investigated C cycle parameters in the two grasslands (*open* = meadow,* shaded* = abandoned grassland). The resistance was calculated as the ratio of parameter performance in drought plots relative to the parameter performance in the control plots during peak drought. The recovery index is a measure for the absolute recovery of the parameter after the end of the drought. *High values* indicate high resistance and recovery, respectively. The *arrow* indicates the shift in resilience caused by abandonment. *ER* ecosystem respiration, *GPP* light-saturated gross primary productivity, *LAI* leaf area index.

To integrate the drought responses across the two grasslands, the resistance and the recovery of studied parameters were related in a bivariate approach (see methods). Drought resistance generally increased from GPP_sat_ to ER to LAI to biomass and was generally lower in the meadow than in the abandoned grassland (Figure 7). In contrast, across all parameters, the recovery index was higher in the meadow than in the abandoned grassland, yielding an overall negative relationship between resistance and recovery.

**Figure 8:**
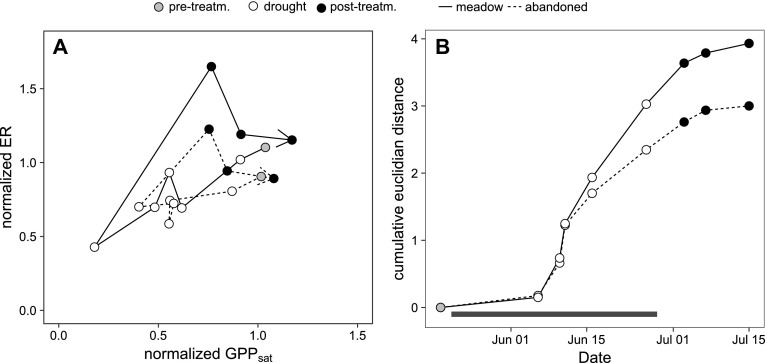
**A** The course of normalized light-saturated rates of gross primary productivity (GPP_sat_) and ecosystem respiration (ER) before (*grey points*), during (*open points*) and after (*black points*) the drought experiment in the meadow (*solid line*) and the abandoned grassland (*dotted line*). Normalized fluxes were calculated as the ratio of the flux in drought monoliths to the respective flux in control monoliths. The direction of the path is given by the *arrow*, *symbols* denote the periods before (*shaded*), during (*open*) and after (*closed*) drought. **B** Cumulative Euclidian distance of the response trajectories of the two grasslands over the course of the drought. The Euclidian distance between two consecutive measurements days is a measure of the system’s change in the bivariate flux space. The cumulative Euclidian distance from beginning of the drought (pretreatment) is a measure of the overall perturbation of the grassland. The *black horizontal bar* indicates period of rain exclusion.

The effect of land-use change on the perturbation of the two CO_2_ flux components GPP_sat_ and ER was assessed in more detail by comparing their drought response trajectories for the meadow and the abandoned grassland. While the trajectories had similar shapes for both grasslands (Figure 8A), the cumulative length of the response trajectory for the meadow was 33% larger than for the abandoned grassland (Figure 8B).

## Discussion

### Does Land Use Alter Drought Resistance and Recovery of Productivity and CO_2_ Fluxes?

The conceptualization of resilience in ecology has led to contrasting definitions and terminologies, which have recently been under some debate (e.g. Hodgson and others 2015; Yeung and Richardson 2016). For analysing the disturbance responses of a system within a given stability domain (“engineering resilience”, sensu Holling 1996), it has been suggested to distinguish resistance and recovery as the two major underlying processes of resilience (Hodgson and others 2015; Nimmo and others 2015; Oliver and others 2015). In our study, we observed that both grasslands were highly resilient to drought, confirming conclusions from earlier studies (Gilgen and Buchmann 2009; Jentsch and others 2011; Hoover and others 2014), though it should be noted that the timing, the magnitude and the interannual pattern of drought may modify specific grassland drought responses (Knapp and others 2015; Estiarte and others 2016; Hoover and Rogers 2016). Furthermore, we found that the role of resistance and recovery for resilience can be strongly altered by land-use change: although the abandoned grassland had a distinctly higher drought resistance, the managed meadow displayed a higher recovery rate across all the studied C cycle parameters. This notion is confirmed when applying a bivariate approach (Figure 7), as recently suggested in the literature (Hodgson and others 2015; Nimmo and others 2015): the abandoned grassland was resilient due to a high resistance, whereas the meadow was less resistant, but resilient due to a high recovery. In consequence, the overall perturbation of the C cycle caused by drought was larger in the managed than the abandoned grassland, as indicated by a greater length of a multivariate response trajectory (Potts and others 2006, Figure 8). This suggests that the loss of ability to recover caused by abandonment was smaller than the concurrent gain of resistance.

The consistent trade-off between resistance and recovery between the two studied grasslands was likely related to differences in plant community composition and associated differences in the prevailing strategies of plant species to cope with drought. Our results suggest that the different responses of the grasslands were not predominantly driven by their relative composition of grasses versus forbs, as the drought and post-drought recovery response of grasses was similar in the meadow and the abandoned grassland. However, the drought response of forbs differed distinctly between the two grasslands: forb biomass was less affected by drought in the abandoned grassland than in the meadow (Figure 3), which contributed to the higher resistance of the abandoned grassland. The forbs in the abandoned grassland were characterized by lower mean SLA compared to the meadow, a trend already previously observed both for SLA and LNC across gradients of decreasing land-use intensity (Bahn and others 1999; Grigulis and others 2013; but note that reduced grazing intensity can also favour species with higher SLA and LNC, see Laliberté and others 2012). Species with lower SLA and LNC are associated with lower growth rates (Lambers and Poorter 1992; Wright and others 2004) and are characterized by a higher tolerance to nutrient stress (Garnier and others 2004; Quétier and others 2007; Grigulis and others 2013). These “conservative species” have been suggested to be more resistant, but less capable of recovering quickly from disturbance (Lambers and Poorter 1992; MacGillivray and Grime 1995; Reich 2014). Conversely, communities dominated by “exploitative species” (fast growth related to a higher SLA and LNC) have been shown to recover better from climatic disturbances (Lepš and others 1982; Grime and others 2000). In our study, all the C cycle parameters studied were more susceptible to drought but recovered more rapidly in the managed meadow, which is more strongly dominated by exploitative species.

Vegetation phenology has been suggested to be sensitive to climate extremes (for example, Jentsch and others 2009). In our study, phenological dynamics during post-drought recovery likely reflected contrasting plant strategies of the two grasslands. The meadow built up new biomass and increased leaf area more rapidly (Figure 2A, G), reflecting its fast-growth strategy. In contrast, the lower necromass in the abandoned grassland at the last sampling date (Figure 2D) indicates a delayed leaf senescence of that plant community in response to the drought. Both grasslands can thereby potentially maintain a higher C uptake later in the season to compensate C deficits from drought (Casper and others 2001).

It has been shown that with increasing time since abandonment the resistance of abandoned fields to drought (Lepš and others 1982) and of shrublands to long-term warming and drought (Kröel-Dulay and others 2015) increases. As secondary succession proceeds, an increasing dominance of woody species (shrubs, trees) also favours plants with more conservative water use, which could buffer effects of dry periods on soil moisture (Teuling and others 2010; Wolf and others 2013; Gavazov and others 2014). As suggested by our results for the water balance (Table S1), such a water-sparing strategy was not yet observed in the abandoned grassland of our study, where herbaceous species prevailed.

### Does Land Use Alter N Uptake During Recovery from Drought?

Site fertility has been shown to modify grassland responses to climate change (Grime and others 2000); however, the immediate role of nutrient availability for ecosystem recovery from drought is still poorly understood. Recent studies have suggested that drought-induced increases in N turnover and availability can increase plant tissue N concentrations (Fuchslueger and others 2014; Canarini and Dijkstra 2015; Arfin Khan and others 2016) and can thereby enhance grassland CO_2_ uptake dynamics during recovery (Roy and others 2016). Our study supports the hypothesis that drought can increase leaf N concentrations during recovery, and indicates that the effect was more pronounced in the managed meadow (Figure 4A, B). This is in line with recent observations that resource pulses can be larger under intensive compared to extensive management (Fuchslueger and others 2014; Schrama and Bardgett 2016) and suggests a higher post-drought availability of N in the meadow compared to the abandoned grassland. Higher rewetting-induced resource pulses in the meadow might also be reflected by a more pronounced stimulation of CO_2_ release from soil (“Birch effect”, Figure 6C, D), which has frequently been associated with a rapid mineralization of organic matter (for example, Borken and Matzner 2009; deVries and others 2012).

Tissue N concentrations result from the uptake of N and its dilution by growth. Since during its recovery from drought, the meadow increased tissue N concentrations while producing more new biomass than the abandoned grassland (Figure 2), it must have taken up distinctly more N. This is confirmed by our ^15^NO_3_ labelling experiment, which suggests a doubling of nitrate uptake in monoliths from the meadow during recovery from drought, while no clear effect was observed for the abandoned grassland (Figure 5). The labelling experiment suggests that increased N uptake on the meadow was not only caused by more pronounced resource pulses, but also by a higher root N uptake capacity, which is typically higher for exploitative, fast-growing species (Osone and others 2008; Grassein and others 2015). Interestingly, on the meadow the additional N taken up during recovery did not remain in the roots, but was used for the production of aboveground biomass. Since the natural abundance δ^15^N of leaves was not affected by drought (Figure 4C, D), it can be assumed that the major sources of N were not strongly altered by drought (Craine and others 2015). Collectively, our results suggest that drought can increase soil N availability and enhance the potential for the regrowth of biomass after drought, which contributed particularly to the rapid recovery on the meadow, where the resource pulse during rewetting was larger and roots had a stronger capacity for N uptake.

## Conclusions

We conclude that the studied grasslands are highly resilient to extreme drought and that land-use change has a strong potential for altering the relative contributions of resistance and recovery to the overall resilience. Our results suggest that abandonment increases the resistance and decreases the recovery of grassland carbon dynamics across different carbon cycle parameters and different measures of resilience. Rapid recovery from drought was supported by drought-induced increases in nitrogen availability and enhanced leaf nitrogen concentrations, which was more pronounced in the managed grassland. We conclude that managed mountain grassland is likely prone to larger overall perturbations from extreme early-summer droughts compared to abandoned grassland. Therefore, ongoing and future land-use changes have the potential to significantly alter impacts of climate extremes on grassland carbon dynamics.

## Electronic Supplementary Material

Below is the link to the electronic supplementary material.

Supplementary material 1 (RTF 22155 kb)
