# Supplementary material for: Land Use Alters the Drought Responses of Productivity and CO2 Fluxes in Mountain Grassland
Source: Ecosystems. 2017 Sep 15;21(4):689–703. doi: 10.1007/s10021-017-0178-0 (PMC5982442; doi:10.1007/s10021-017-0178-0)
Supplement: Supplementary file 1 — Supplementary material 1 (RTF 22155 kb) [file 10021_2017_178_MOESM1_ESM.rtf]

Supplementary Figures and Tables

Fig S1. Net ecosystem CO2 flux in relation to photon flux density (PFD) in monoliths from the meadow and the abandoned grassland subjected to ambient conditions (open symbols) and drought (closed symbols) . The light response curves were estimated by fitting a rectangular hyperbolic model (Ruimy and others 1995) to the data obtained for the control (solid line) and the drought (dotted line) treatments, respectively . The vertical dashed line represents the threshold of PFD above which CO2 exchange was defined as light saturated (GPPsat). 


Fig S2. Phytomass of flowers, leaves and stems [g m-2] of monoliths from the meadow and the abandoned grassland subjected to ambient conditions (open symbols) and drought (closed symbols) in the meadow and the abandoned grassland and sampled at the end of drought (1 July) and during recovery (24 July, 22 August). Error bars indicate standard errors of the mean (n=3 for July and n=4 for August samplings), stars indicate significant treatment effects within land use and sampling date (p-value: *** < 0.001 < ** < 0.01 < * < 0.05 < (*) < 0.1).


Fig S3. Leaf biomass of bulk samples, forbs and grasses [g m-2] of monoliths from the meadow and the abandoned grassland subjected to ambient conditions (open symbols) and drought (closed symbols)  and sampled at the end of drought (1 July) and during recovery (24 July, 22 August). Error bars indicate standard errors of the mean (n=3 for July and n=4 for August samplings), stars indicate significant treatment effects within land use and sampling date (p-value: *** < 0.001 < ** < 0.01 < * < 0.05 < (*) < 0.1).


Table S1: Water balance during the rain-exclusion period.
	Treatment	Precipitation	Leachates	∆ Soil Water	Evapotranspiration	
meadow	Control	180	4 ± 1	-6 ± 3	182 ± 4	
 	Drought	0	0	-72 ± 10	72 ± 10	
abandoned	Control	169	5 ± 0.2	4 ± 4	160 ± 5	
 	Drought	0	0	-67 ± 9	67 ± 9	
Water addition corresponds to the amount of water manually added during the drought period. Errors denote standard error of the mean (n = 3-5).


Table S2: Drought and land-use effects on phytomass of plant organs.
Phytomass	 Campaign	Weeks after rewetting	Treatment	Land use	Treatment * Land use	
	Resistance	0	***	n.s.	(*)	
leaves	Recovery 1	3.5	*	n.s.	n.s.	
 	Recovery 2	8	n.s. 	n.s.	n.s.	
reproductive 
organs	Resistance	0	n.s.	n.s.	n.s.	
	Recovery 1	3.5	*	n.s.	n.s.	
	Recovery 2	8	 n.s.	n.s. 	 n.s.	
	Resistance	0	n.s.	n.s.	n.s.	
stem	Recovery 1	3.5	**	(*)	n.s.	
 	Recovery 2	8	(*)	 n.s.	 n.s.	
Permutational ANOVA testing overall treatment effect, land-use effect and their interaction on phytomass of plant organs. Resistance = 1 July, peak drought, Recovery 1 = 24 July, Recovery 2 = 22 August. Stars indicate the significance level: *** < 0.001 < ** < 0.01 < * < 0.05 < (*) < 0.1.
Permutational ANOVA testing overall treatment effect, land-use effect and their interaction on phytomass of plant organs. Resistance = 1 July, peak drought, Recovery 1 = 24 July, Recovery 2 = 22 August. Stars indicate the significance level: *** < 0.001 < ** < 0.01 < * < 0.05 < (*) < 0.1.


Table S3: Drought and land-use effects on leaf biomass of functional groups.
Biomass of leaves	Weeks after rewetting	Treatment	Land use	Treatment * Land use	
	Resistance	0	***	n.s.	(*)	
Bulk	Recovery 1	3.5	**	n.s.	n.s.	
 	Recovery 2	8	 n.s.	 n.s.	 n.s.	
	Resistance	0	n.s.	***	*	
Forbs	Recovery 1	3.5	**	***	*	
 	Recovery 2	8	*	***	*	
	Resistance	0	***	n.s.	n.s.	
Grasses	Recovery 1	3.5	n.s.	n.s.	n.s.	
 	Recovery 2	8	 n.s.	 n.s.	 n.s.	
Permutational ANOVA testing overall treatment effect, land-use effect and their interaction on leaf biomass of community samples, forbs and grasses. Resistance = 1 July, peak drought, Recovery 1 = 24 July, Recovery 2 = 22 August. Stars indicate the significance level: *** < 0.001 < ** < 0.01 < * < 0.05 < (*) < 0.1.
